# 1050. Phase 3 Trial to Evaluate the Safety, Tolerability, and Immunogenicity of V114 Followed by 23valent Pneumococcal Polysaccharide Vaccine 6 Months Later in At-risk Adults Aged 18–49 Years (PNEU-DAY): A Subgroup Analysis by Baseline Risk Factors

**DOI:** 10.1093/ofid/ofab466.1244

**Published:** 2021-12-04

**Authors:** Laura Hammitt, Dean Quinn, Ewa Janczewska, Francisco J Pasquel, Richard Tytus, K Rajender Reddy, Katia Abarca, Ilsiyar M Khaertynova, Ron Dagan, Rachel Dawson, Jennifer McCauley, Kyeongmi Cheon, Alison Pedley, Tina Sterling, Gretchen Tamms, Luwy Musey, Ulrike K Buchwald

**Affiliations:** 1 Johns Hopkins, Baltimore, MD; 2 P^3^ Research, Wellington Clinical Trial Research Unit, Wellington, Wellington, New Zealand; 3 The School of Health Sciences in Bytom, Medical University of Silesia, Bytom, Slaskie, Poland; 4 Emory, University School of Medicine, Atlanta, Georgia; 5 McMaster University, Hamilton, Ontario, Canada; 6 Perelman School of Medicine, University of Pennsylvania, Philadelphia, Pennsylvania; 7 Escuela de Medicina, Pontificia Universidad Catolica de Chile, Santiago, Region Metropolitana, Chile; 8 Department of Infectious Diseases, Kazan State Medical Academy, Kazan, Tatarstan, Russia; 9 Ben-Gurion University of the Negev, Beer Sheva, HaDarom, Israel; 10 Merck & Co., Inc., Kenilworth, New Jersey

## Abstract

**Background:**

Risk factors (RFs) for pneumococcal disease (PD) in immunocompetent individuals include comorbidities, behavioral habits, or living in a community with increased risk of PD transmission. RF stacking of comorbidities is associated with a higher incidence of PD, approaching that of immunocompromised individuals. Pneumococcal vaccination of certain adults is recommended with the 23-valent pneumococcal polysaccharide vaccine (PPSV23) alone/sequentially with pneumococcal conjugate vaccine (PCV). V114, an investigational 15-valent PCV, contains 2 epidemiologically important serotypes (STs), 22F and 33F, in addition to the 13 STs in 13-valent PCV (PCV13).

**Methods:**

PNEU-DAY was a Phase 3 study evaluating V114 or PCV13 administered on Day 1, and PPSV23 given 6 months later, in adults aged 18–49 years with or without RFs. This subgroup analysis assessed safety, tolerability, and immunogenicity of V114 and PCV13 based on the number of baseline PD RFs, which included chronic liver, lung, and heart disease, diabetes mellitus, tobacco use, and alcohol consumption. Adverse events (AEs; overall and solicited) were collected after each vaccination. Immunogenicity assessment was based on ST-specific opsonophagocytic activity (OPA) at 30 days after each vaccination. Subgroup analyses were conducted by RF group (0, 1, or ≥2 RFs for PD).

**Results:**

Among the 1515 participants randomized to V114 (n=1135) or PCV13 (n=380), 25.2% had no RFs, 54.7% had 1 RF and 20.1% had ≥2 RFs for PD at baseline. The proportions of participants with solicited AEs following V114/PCV13 and PPSV23 were comparable across the 3 subgroups, with injection-site pain, myalgia, and fatigue being the most common. V114 and PCV13 were immunogenic in all subgroups based on OPA geometric mean titers (GMTs) at 30 days post-vaccination for the 13 shared STs (**Figure**); in addition, V114 induced a robust immune response to the 2 unique STs (22F, 33F) in all subgroups. PPSV23 following PCV was immunogenic for all 15 STs contained in V114 across all subgroups.

Figure. Serotype-specific OPA GMTs at baseline and 30 days post-vaccination with V114 and PCV13 by number of baseline risk factors (per-protocol population)

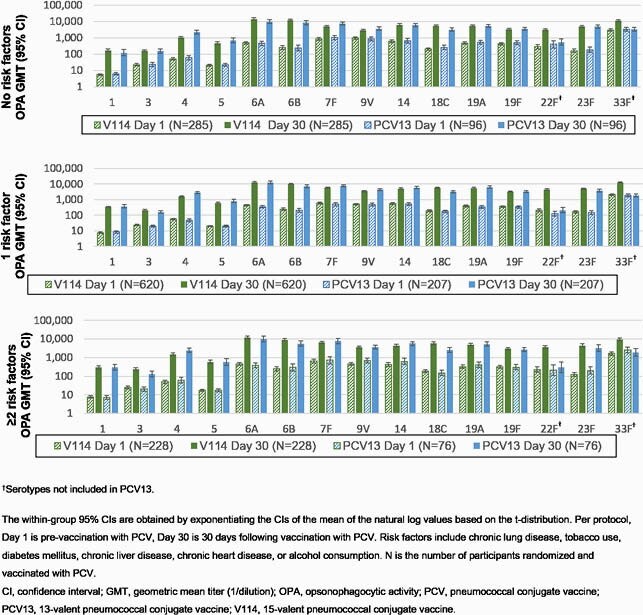

**Conclusion:**

V114 administered alone/sequentially with PPSV23 is well tolerated and immunogenic for all 15 vaccine STs, including those not contained in PCV13, in immunocompetent adults aged 18–49 years, regardless of the number of baseline RFs.

**Disclosures:**

**Laura Hammitt, MD**, **MedImmune** (Grant/Research Support, Scientific Research Study Investigator, Research Grant or Support)**Merck & Co., Inc.** (Grant/Research Support, Scientific Research Study Investigator, Research Grant or Support)**Novavax** (Grant/Research Support, Scientific Research Study Investigator, Research Grant or Support)**Pfizer** (Grant/Research Support, Scientific Research Study Investigator, Research Grant or Support) **Francisco J. Pasquel, MD, MPH**, **Boehringer Ingelheim** (Consultant)**Dexcom** (Grant/Research Support)**Eli Lilly & Company** (Consultant)**Insulet** (Grant/Research Support)**Merck & Co., Inc.** (Consultant, Grant/Research Support) **K. Rajender Reddy, MD**, **BMS** (Grant/Research Support)**Deciphera** (Advisor or Review Panel member)**Gilead** (Grant/Research Support)**Grifols** (Grant/Research Support)**HCC-TARGET** (Grant/Research Support)**Intercept** (Grant/Research Support)**Mallinckrodt** (Grant/Research Support, Advisor or Review Panel member)**NASH-TARGET** (Grant/Research Support)**Pfizer** (Advisor or Review Panel member)**Sequana** (Grant/Research Support) **Ron Dagan, MD**, **Medimmune/AstraZeneca** (Grant/Research Support, Scientific Research Study Investigator, Research Grant or Support)**MSD** (Consultant, Grant/Research Support, Scientific Research Study Investigator, Advisor or Review Panel member, Research Grant or Support, Speaker’s Bureau)**Pfizer** (Consultant, Grant/Research Support, Scientific Research Study Investigator, Advisor or Review Panel member, Research Grant or Support, Speaker’s Bureau) **Rachel Dawson, D.O. MPH**, **Merck & Co., Inc.** (Employee, Shareholder) **Jennifer McCauley, BSc**, **Merck & Co., Inc.** (Employee) **Kyeongmi Cheon, Ph.D.**, **Merck & Co., Inc.** (Employee, Shareholder) **Alison Pedley, PhD**, **Merck & Co., Inc.** (Employee) **Tina Sterling, BS**, **Merck & Co., Inc.** (Employee, Shareholder) **Gretchen Tamms, B.S.**, **Merck Sharp and Dohme** (Employee, Shareholder) **Luwy Musey, MD**, **Merck & Co., Inc.** (Employee) **Ulrike K. Buchwald, MD, MS**, **Merck & Co., Inc.** (Employee)**TB Alliance** (Employee)

